# Krüppel-like factor 4 expression in oral carcinoma cells and hypermethylation at the gene promoter

**DOI:** 10.1186/s12903-016-0172-5

**Published:** 2016-02-04

**Authors:** Ayumi Yamaguchi, Karen Kuroyama, Ayana Tokura, Atsushi Saito, Huhga Arikawa, Takahisa Hasebe, Dai Usui, Kosuke Yamaguchi, Tadashige Chiba, Kazushi Imai

**Affiliations:** School of Life Dentistry at Tokyo, The Nippon Dental University, 1-9-20, Chiyoda-ku, Fujimi, Tokyo 102-8159 Japan; Department of Biochemistry, School of Life Dentistry at Tokyo, The Nippon Dental University, 1-9-20 Chiyoda-ku, Fujimi, Tokyo 102-8159 Japan

**Keywords:** Gene promoter, Hypermethylation, Krüppel-like factor, Oral cancer

## Abstract

**Background:**

Krüppel-like factor 4 (KLF4) is a transcription factor regulating proliferation-differentiation balance of epithelium, and down-regulated in less-differentiated and advanced oral carcinomas. Although the expression is inactivated by the promoter hypermethylation in malignant tumor cells, it remains unknown in oral carcinoma cells.

**Methods:**

Genomic DNA isolated from nine different oral carcinoma cell lines and a normal keratinocyte line was treated with sodium bisulfite, and methylation at *KLF4* gene promoter was determined by PCR direct-sequence analysis. KLF4 expression in cells cultured with or without demethylation reagent was monitored by quantitative real-time PCR and immunoblot.

**Results:**

A 237-bp promoter region spanning − 718 and − 482 of *KLF4* gene was hypermethylated in oral carcinoma cells that express *KLF4* at a low level, but the methylation was infrequent in cells expressing *KLF4* high amount. The downstream region from − 481 to +192 was not methylated in any cell lines. Demethylation treatment of cells up-regulated the expression at mRNA and protein levels.

**Conclusion:**

This study demonstrated that hypermethylation at a narrow range of the promoter region down-regulates KLF4 expression, and suggests that the loss of expression by the hypermethylation contributes to oral carcinoma progression.

**Electronic supplementary material:**

The online version of this article (doi:10.1186/s12903-016-0172-5) contains supplementary material, which is available to authorized users.

## Background

Oral carcinomas are a most frequent malignant tumor of the head and neck, but the patients’ prognosis has not been improved sufficiently. Carcinoma cells at invasive front frequently show aberrant gene expression, and dedifferentiate into mesenchymal cell-like states in a process of disease progression [1–3]. In addition, sustained proliferation is another prominent feature in an aggressive subset of carcinomas. Proliferation and differentiation balance of cells control development and homeostasis of epithelium and play a definitive role in pathological state of carcinomas [4]. They are largely regulated by tumor suppressive gene, and expression of the genes is frequently inactivated by epigenetic mechanisms [5]. It is important to unveil a cause inactivating tumor suppressive gene expression to understand mechanisms of oral carcinoma progression.

In stratified squamous epithelium including oral epithelium, krüppel-like factor (KLF) 4 and 5 are expressed in post-mitotic keratinizing suprabasal cells and proliferative less-differentiated basal cells, respectively. They critically control a proliferation-differentiation balance of the epithelium [6, 7]. KLFs transcriptionally regulate target gene expression through binding on the promoter, and previous studies documented that loss of KLF4 expression associates with dedifferentiation of oral carcinoma cells and is frequently observed in an aggressive subset of the carcinomas [7, 8]. It suggests an involvement of the loss of expression in carcinoma progression.

Since chromosomal deletion of *KLF4* gene locus at 9q31.2 is a rare event in carcinomas [9, 10], epigenetic inactivation of the gene is a prime candidate responsible for the loss of expression. Among epigenetic aberrations found in carcinoma cells, promoter hypermethylation is a most common causative to inactivate gene expression [11, 12]. In fact, hypermethylation at *KLF4* gene promoter and enhancer is documented in carcinomas of the colon, stomach, cervix and kidney [13–16]. However, the hypermethylated regions are variably localized in carcinomas of different origin and unknown in oral carcinomas. In this study, we analyzed the hypermethylation and correlation with KLF4 expression in oral carcinoma cells.

## Methods

### Cell lines

Oral carcinoma cell lines (Ca9.22, Ho-1-u-1, HOC313, HSC2, HSC3, KOSC3, OSC19, SCCKN, TSU) and an immortalized but not transformed normal keratinocyte cell line, HaCaT [17], were cultured in 10 % fetal bovine serum-containing medium.

### Bisulfite-modified sequence analysis of *KLF4* promoter region

Promoter methylation states at *KLF4* gene were analyzed according to a previous study [18]. Genomic DNA isolated from cells were treated with sodium bisulfite and applied for PCR amplification of the promoter region spanning − 718 and +192 (the transcription start site was set as +1) for direct-sequence analysis. The primer sequences used for the analysis are as follows: 5’-^−736^GTATGTTAGTAGGGGTG-3’ (forward), 5’-^−442^GAGTTTGTTGATTTAGTTGT-3’ (forward), 5’-^−331^AAGGAAGTTATAAGTAAGGAA-3’ (forward), 5’-^−72^AATAAAACTAACTACC-3’ (reverse), and 5’-^+213^AAACCCAAAACCCCAAATTAA-3’ (reverse). We referred a DNA sequence data of *KLF4* gene deposited to GenBank (DQ658241.1).

### Quantitative real-time PCR

Total RNA isolated form cells with or without 5 μM 5-aza-2-deoxycitidine (5-aza) treatment was reverse transcribed into cDNA by MultiScribe Reverse Transcriptase (Applied Biosystems) and subjected to quantitative real-time PCR using the StepOne Real-time PCR system (Applied Biosystems). PCR conditions were 95 °C for 20 s followed by 40 cycles of 95 °C for 1 s and 60 °C for 20 s. The TaqMan probes specific to *KLF4* (Hs00358836_m1, Applied Biosystems) was used. Expression levels normalized against *ACTB* (TaqMan Endogenous Control Human ACTB, Applied Biosystems) were calculated by the standard curve method (2^-∆∆Ct^). To analyze relative-fold of changes of the expression, the expression after the 5-aza treatment was divided by that without treatment.

### Immunoblot

Total cell lysates in SDS sample buffer containing 1 mM phenylmethylsulfonyl fluoride and protease inhibitor cocktail (Roche Diagnostic GmbH) was applied to SDS-polyacrylamide gels under the reducing condition, and electrotransferred to PVDF membranes. The membranes were probed with antibodies against KLF4 (Santa Cruz Biotechnology) or β-actin (Sigma-Aldrich) followed by horseradish peroxidase-conjugated secondary antibodies. The signals were detected using Chemi-Lumi One Super (Nakarai Tesque) and captured on Ez-Capture MG (ATTO).

## Results

### Expression of KLF4 in oral carcinoma cells

**Fig. 1:**
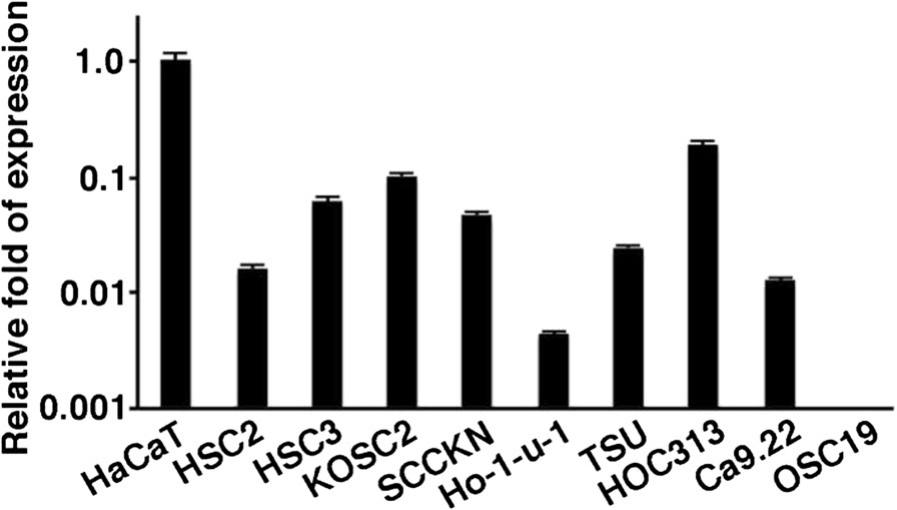
Expression of *KLF4* mRNA in oral carcinoma cell lines and normal keratinocytes (HaCaT). *KLF4* expression was quantitatively examined by the real-time PCR. Relative expression was standardized by expression of *ACTB* in each sample (*n* = 4) and compared with the expression in HaCaT cells

Expression of *KLF4* mRNA was quantified by the real-time PCR (Fig. 1). Among carcinoma cell lines, it was strongly expressed in HaCaT normal keratinocytes. It was detected at a relatively high level in KOSC2 cells and HOC313 cells, low in HSC2 cells, Ho-1-u-1 cells and Ca9.22 cells, and undetectable in OSC19 cells.

### Promoter hypermethylation in carcinoma cells

**Fig. 2:**
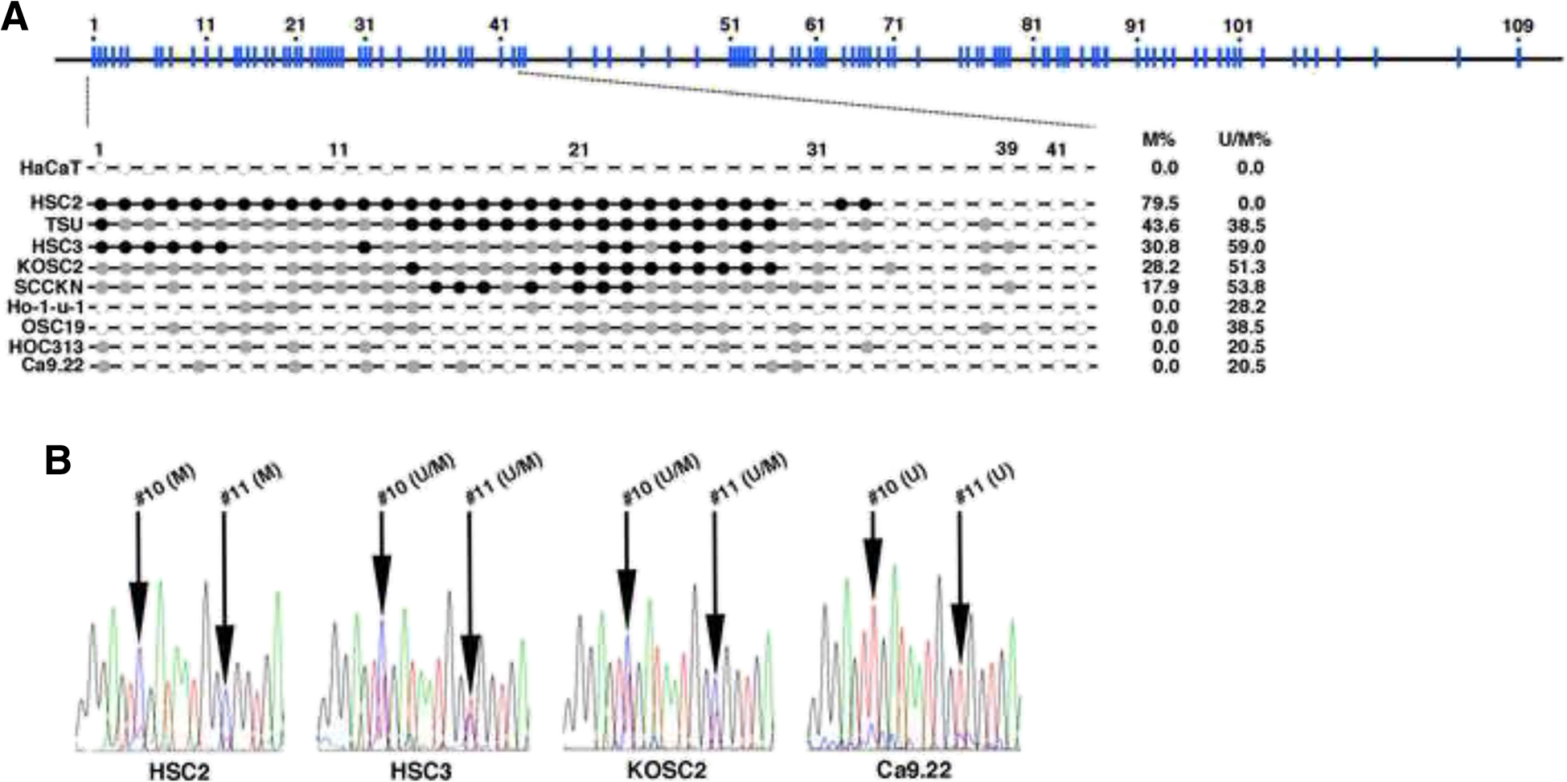
Methylation of *KLF4* gene promoter. **a** Position of methylation-susceptible cytosines in the promoter is indicated as blue (*upper line*) and the cytosines are numerically numbered from #1 to #109. Lower lines show cytosines that were entirely methylated (*black*) or unmethylated (*white*) and mixture of methylation and unmethylation (*gray*). M% and U/M% indicate percentage of methylated cytosines and the mixture in 39 cytosines, respectively. **b** Sequence data on #10 and #11 cytosines. Four carcinoma cell lines containing methylated (M) and unmethylated (U) cytosines and the mixture (U/M) were presented as an example

**Fig. 3:**
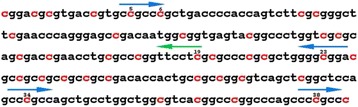
Transcription factor binding sites in the 237-bp region. Methylation-susceptible sites are marked by red, and Sp1-binding sites (*blue arrows*) and PU.1-binding site (*green line*) are indicated

Methylation at *KLF4* gene promoter from − 718 to +192 was examined by the bisulfite-modified PCR direct-sequence analysis. The promoter included 109 methylation-susceptible cytosines (Additional file 1: Figure S1), and we described methylation state of each cytosine as U (unmethylated), M (methylated) or U/M (mixture of unmethylation and methylation) in this study. In contrast to the absence of methylation in HaCaT cells, all carcinoma cell lines contained M and/or U/M cytosines and frequency of M cytosine was largely different (Fig. 2 and Additional file 1: Table S1). The methylation was confined in a 237-bp region spanning − 718 and − 482 that contains 39 methylation-susceptible cytosines designated as #1 to #39, and the cytosines in 673-bp region downstream of #39 cytosine (#40-#109) was not methylated. Computational analysis indicated that the 237-bp region contains methylation-susceptible cytosines at four Sp1-binding sites (#5, 6, 23, 34 and 38 cytosines) and one PU.1-binding site (#19 cytosine). The #5, 6, 19 and 23 cytosines were frequently methylated in cells that expressed *KLF4* at low level (Fig. 3 and Additional file 1: Table S1).

### *KLF4* expression after the demethylation

**Fig. 4:**
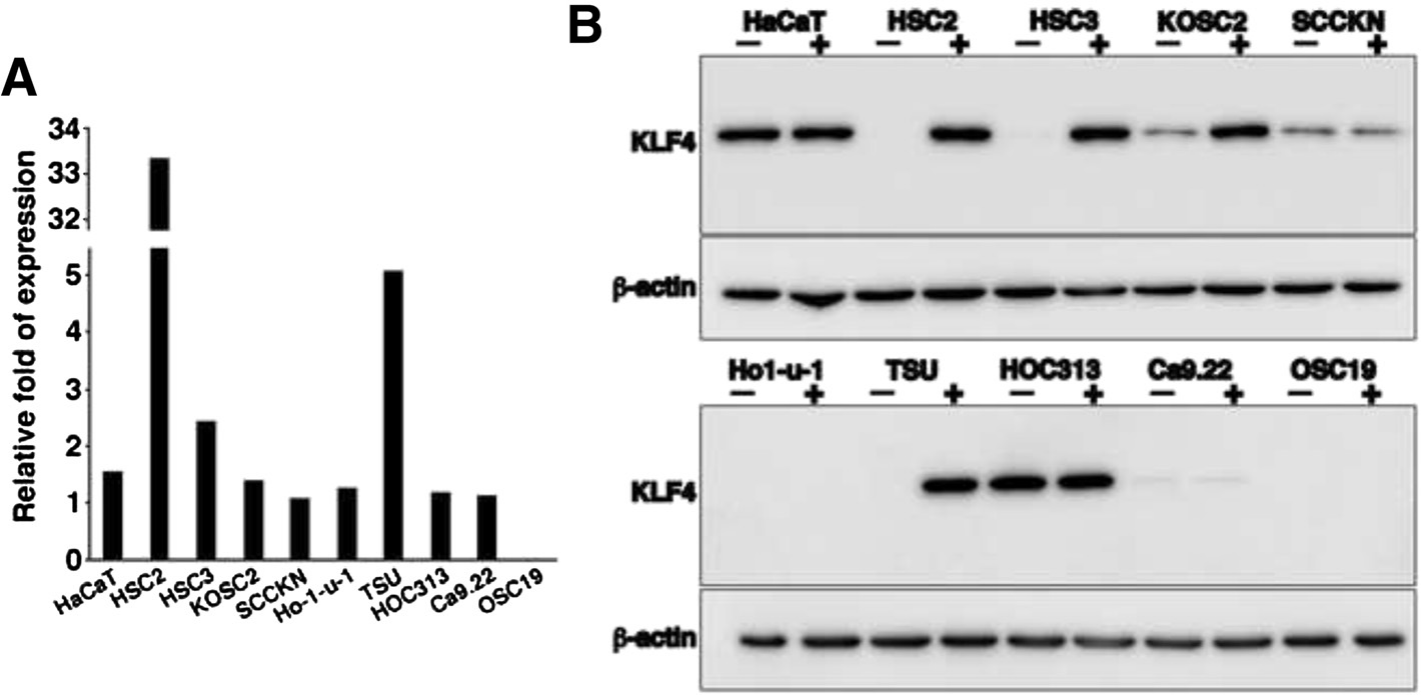
Expression of KLF4 after the demethylation. **a** Relative fold of *KLF4* mRNA expression in cells treated with 5-aza demethylation reagent divided by the expression without 5-aza treatment (*n* =4). **b** KLF4 protein expression in cells with (+) and without (−) 5-aza treatment were examined by immunoblot. β-actin was used as an internal control

To examine an involvement of the methylation in KLF4 expression, cells were treated with a demethylation reagent 5-aza and the expression was analyzed at mRNA and protein levels. The treatment strongly increased mRNA expression in TSU cells and especially HSC2 cells (Fig. 4a) in which the promoter were hypermethylated extensively. Other cell lines increased the expression variously, excluding OSC19 cells that expressed mRNA below a detectable level. KLF4 protein expression was almost identical to the mRNA expression. It was undetectable in HSC2 cells, HSC3 cells, Ho-1-u-1 cells, TSU cells and OSC19 cells. After the demethylation treatment, hypermethylated HSC2 cells and TSU cells strongly up-regulated the protein expression (Fig. 4b), and Ho-1-u-1 cells, HOC313 cells and Ca9.22 cells that contained no M cytosine did not increase the expression. OSC19 cells did not express the protein like mRNA. Among three cell lines containing both M and U/M cytosines in various rates, HSC3 cells and KOSC2 cells apparently increased the expression but another (SCCKN cells) did not.

## Discussion

Carcinoma cells acquire aggressive behaviors in a process of progression by activating and inactivating tumor-associated gene expression. KLF4 expression is decreased in many types of carcinomas in advanced stage and the loss of expression initiates epithelial-mesenchymal transition of carcinoma cells that strongly stimulates the progression [19]. In fact KLF4 is down-regulated in less-differentiated and advanced oral carcinomas and suppresses dedifferentiation of the cells [7]. Although the promoter hypermethylation is considered as a cause of the loss of expression [13–16], it is not known in oral carcinomas. This study shows that the expression closely correlates with hypermethylation states at a 239 bp-region in the promoter.

Hypermethylation responsible for gene inactivation is frequently observed in CpG islands of tumor suppressive genes [11, 12], and it was shown in *KLF4* gene in this study, suggesting that the region is important to regulate KLF4 expression in oral carcinoma cells. This is supported by that demethylation treatment up-regulated KLF4 mRNA and protein in the hypermethylated cells but not the cells without hypermethylation. Ca9.22 cells, Ho-1-u-1 cells and OSC19 cells that were not hypermethylated almost did not increase the expression. It suggests a presence of additional factors to activate the expression. However, it is clear that the hypermethylation is responsible for KLF4 down-regulation, and that the 237 bp-region may play an important role in the expression.

Hypermethylation of *KLF4* gene is happened at enhancer of the gene in human malignant tumor cells; −2,128 ~ −1,770 region in medulloblastoma cells [20] and − 1,852 ~ −1,658 region in cervical carcinoma cells [15]. Although we did not addressed methylation at the enhancer in this study, methylation at promoter region is more directly as a platform to regulate gene expression in general [11, 12]. Hypermethylation at *KLF4* gene promoter has been observed in gastric carcinoma cells (−130 ~ −13 region; ref. 14) and in colorectal carcinoma cells (+79 ~ +355 region; ref. 13), but the − 481 ~ +192 region was not methylated in this study. It indicates that hypermethylation restricted at a 237-bp region from − 718 to − 482 is important for the negative regulation in oral carcinoma cells. Recent evidences established that hypermethylation occurs at different region of gene promoter/enhancer in different types of carcinoma cells and the promoter hypermethylation is more designate for cell-lineage and cell-type specification [11, 12]. It is plausible that the hypermethylation-sensitive sites in oral carcinoma cells locate apart from other-types of carcinoma cells.

The 237-bp region encodes Sp1 and PU.1 binding sites that are required for the expression [21, 22]. Sp1 plays a critical role in epithelial development [23, 24], and *Klf4*^*−/−*^ mice develop oral carcinomas [8, 25]. Therefore, hypermethylation at the 237-bp region appears to have a significant role in KLF4 down-regulation. Since loss of KLF4 expression closely associates with oral carcinoma progression, restoring the expression may be an intriguing strategy to treat the patients.

## Conclusions

This study demonstrated that *KLF4* gene promoter was hypermethylated in oral carcinoma cells at a different region from other-types of carcinoma cells, and that it was associated with KLF4 expression. Since KLF4 is tumor suppressive, its inactivation by the promoter hypermthylation may be a mechanism of oral carcinoma progression as in carcinoma cell lines analyzed in this study.

## Additional file

Additional file 1: Figure S1. Methylation susceptible sites at *KLF4* gene and the promoter. DNA sequence analyzed in this study is shown (the sequence in exon 1 is capitalized). Cytosines potentially susceptible to methylation and their numerical number were highlighted in red, and a hypermethylated 237-bps region was underlined. **Table S1.** A summary of methylation states at each methylation susceptible cytosine. (PDF 160 kb)

## Abbreviations

5-aza: 5-aza-2-deoxycitidine
KLF: Krüppel-like factor
M cytosine: Methylated cytosine
U cytosine: Unmethylated cytosine
U/M cytosine: Mixture of unmethylated and methylated cytosines
