# Supplementary material for: Krüppel-like factor 4 expression in oral carcinoma cells and hypermethylation at the gene promoter
Source: BMC Oral Health. 2016 Feb 4;16:13. doi: 10.1186/s12903-016-0172-5 (PMC4743192; doi:10.1186/s12903-016-0172-5)
Supplement: Additional file 1: Figure S1. — Methylation susceptible sites at KLF4 gene and the promoter. DNA sequence analyzed in this study is shown (the sequence in exon 1 is capitalized). Cytosines potentially susceptible to methylation and their numerical number were highlighted in red, and a hypermethylated 237-bps region was underlined. Table S1. A summary of methylation states at each methylation susceptible cytosine. (PDF 160 kb) [file 12903_2016_172_MOESM1_ESM.pdf]

```
       1 2 3 4 5 6 7 8 9
-718   cggacgcg tgaccgtgcc gcccgctgac cccaccagtc ttcgcgggct tcgaacccag
       10 11 12 13 14 15 16 17 18
-660   ggagccgaca atggcggtga gtacggccct ggtcgcgcag cgaccgaacc tgcgcccggt
       19 20 21 22 23 24 25 26 27 28 29 30 31 32
-600   tcctcgcgcc ccgcgctggg gcggacgccg ccgccgccgc cgacaccact gccgccggcg
       33 34 35 36 37 38
-540   tcagctcggc tccagcccgc cagctgcctg gctggcgtca cggcccggcc cagccccgcc
       39 40 41 42 43 44
-480   cgcgcccctc cttcccctcc cccgccccca cgtgcgccga gtttgttgat ttagctgcca
       45 46 47 48
-420   tagcaacgat ggaagggagc ctcggggggg gcggagagaa gaaagggagg ggcggggcat
       49 50 51 52 53 54
-360   gggagaaggc ggaggaaaag gctgtagcga aggaagttat aagtaaggaa cgcgcgccgg
       55 56 57 58 59 60 61 62
-300   cggccggcag tttcccgacc agagagaacg aacgtgtctg cgggcgcgcg gggagcagag
       64 65 66 67 68 69 70 71 72
-240   gcggtggcgg gcggcggcgg caccgggagc cgccgagtga ccctccccgc cccgcccccc
       73 74 75 76 77 78 79 80
-180   acctctgctc ccacccgccc gtggcccgcg cccatggccg cgcgcgctcc acacaactca
       81 82 83 84 85 86 87 88 89 90
-120   ccggagtccg cgccttgcgc cgccgaccag ttcgcagctc cgcgccacgg cagccagtct
       91 92 93 94 95 96 97
-60    cacctggcgg caccgcccgc ccaccgcccc ggccacagcc cctgcgccca cggcagcact
       98 99 100 101 102 103 104
+1     CGAGGCGACC GCGACAGTGG TGGGGGACGC TGCTGAGTGG AAGAGAGCGC AGCCCGGCCA
       105 106 107
+61    CCGGACCTAC TTACTCGCCT TGCTGATTGT CTATTTTTGC GTTTACAACT TTTCTAAGAA
       108
+121   CTTTTGTATA CAAAGGAACT TTTTAAAAAA GACGCTTCCA AGTTATATTT AATCCAAAGA
       109
+181   AGAAGGATCT CGG
```

Fig. S1. Methylation susceptible sites at *KLF4* gene and the promoter. DNA sequence analyzed in this study is shown (the sequence in exon 1 is capitalized). Cytosines potentially susceptible to methylation and their numerical number were highlighted in red, and a hypermethylated 237-bps region was underlined.

Table S1. A summary of methylation states at each methylation susceptible cytosine.

cytosine #	HaCaT	HSC2	HSC3	KOSC2	HOC313	Ca9.22	SCCKN	HO-1-u-1	TSU	OSC19
1	U	M	M	U/M	U/M	U/M	U/M	U	M	U
2	U	M	M	U/M	U	U	U/M	U	U/M	U
3	U	M	M	U/M	U	U	U	U	U/M	U
4	U	M	M	U/M	U	U	U/M	U	U	U/M
5	U	M	M	U/M	U	U/M	U	U	U/M	U
6	U	M	M	U/M	U	U	U	U	U/M	U/M
7	U	M	U/M	U/M	U/M	U	U/M	U/M	U/M	U/M
8	U	M	U/M	U	U	U	U	U/M	U/M	U/M
9	U	M	U/M	U/M	U/M	U/M	U/M	U/M	U/M	U
10	U	M	U/M	U/M	U	U	U/M	U	U/M	U
11	U	M	U/M	U/M	U	U	U/M	U	U/M	U
12	U	M	M	U/M	U/M	U/M	U/M	U	U/M	U
13	U	M	U/M	U/M	U	U	U/M	U/M	U/M	U/M
14	U	M	U/M	M	U	U/M	U/M	U/M	M	U/M
15	U	M	U/M	U/M	U	U	M	U	M	U
16	U	M	U/M	U/M	U	U/M	M	U	M	U
17	U	M	U/M	U/M	U	U	M	U	M	U
18	U	M	U/M	U/M	U	U	U/M	U	M	U
19	U	M	U/M	U/M	U	U	M	U/M	M	U
20	U	M	U/M	M	U	U	U/M	U	M	U
21	U	M	U/M	M	U/M	U	M	U/M	M	U/M
22	U	M	M	M	U	U	M	U	M	U/M
23	U	M	M	M	U	U	M	U/M	M	U/M
24	U	M	U/M	M	U	U	U/M	U/M	M	U/M
25	U	M	M	M	U	U	U/M	U/M	M	U/M
26	U	M	M	M	U	U	U/M	U/M	M	U/M
27	U	M	U/M	M	U/M	U	U/M	U	M	U/M
28	U	M	M	M	U	U	U/M	U	M	U
29	U	M	U/M	M	U	U/M	U/M	U	M	U
30	U	U	U/M	U	U/M	U/M	U/M	U	U/M	U/M

31	U	U	U/M	U/M	U	U	U/M	U	U/M	U
32	U	M	U/M	U	U	U	U	U	U	U
33	U	M	U/M	U	U/M	U	U	U	U/M	U
34	U	U	U	U/M	U	U	U	U	U	U
35	U	U	U	U	U	U	U	U	U	U
36	U	U	U	U	U	U	U	U	U	U
37	U	U	U	U	U	U	U	U	U	U
38	U	U	U/M	U/M	U	U	U	U	U/M	U/M
39	U	U	U/M	U	U	U	U/M	U	U	U
40	U	U	U	U	U	U	U	U	U	U
41	U	U	U	U	U	U	U	U	U	U
42	U	U	U	U	U	U	U	U	U	U
43	U	U	U	U	U	U	U	U	U	U
44	U	U	U	U	U	U	U	U	U	U
45	U	U	U	U	U	U	U	U	U	U
46	U	U	U	U	U	U	U	U	U	U
47	U	U	U	U	U	U	U	U	U	U
48	U	U	U	U	U	U	U	U	U	U
49	U	U	U	U	U	U	U	U	U	U
50	U	U	U	U	U	U	U	U	U	U
51	U	U	U	U	U	U	U	U	U	U
52	U	U	U	U	U	U	U	U	U	U
53	U	U	U	U	U	U	U	U	U	U
54	U	U	U	U	U	U	U	U	U	U
55	U	U	U	U	U	U	U	U	U	U
56	U	U	U	U	U	U	U	U	U	U
57	U	U	U	U	U	U	U	U	U	U
58	U	U	U	U	U	U	U	U	U	U
59	U	U	U	U	U	U	U	U	U	U
60	U	U	U	U	U	U	U	U	U	U
61	U	U	U	U	U	U	U	U	U	U
62	U	U	U	U	U	U	U	U	U	U

63	U	U	U	U	U	U	U	U	U	U
64	U	U	U	U	U	U	U	U	U	U
65	U	U	U	U	U	U	U	U	U	U
66	U	U	U	U	U	U	U	U	U	U
67	U	U	U	U	U	U	U	U	U	U
68	U	U	U	U	U	U	U	U	U	U
69	U	U	U	U	U	U	U	U	U	U
70	U	U	U	U	U	U	U	U	U	U
71	U	U	U	U	U	U	U	U	U	U
72	U	U	U	U	U	U	U	U	U	U
73	U	U	U	U	U	U	U	U	U	U
74	U	U	U	U	U	U	U	U	U	U
75	U	U	U	U	U	U	U	U	U	U
76	U	U	U	U	U	U	U	U	U	U
77	U	U	U	U	U	U	U	U	U	U
78	U	U	U	U	U	U	U	U	U	U
79	U	U	U	U	U	U	U	U	U	U
80	U	U	U	U	U	U	U	U	U	U
81	U	U	U	U	U	U	U	U	U	U
82	U	U	U	U	U	U	U	U	U	U
83	U	U	U	U	U	U	U	U	U	U
84	U	U	U	U	U	U	U	U	U	U
85	U	U	U	U	U	U	U	U	U	U
86	U	U	U	U	U	U	U	U	U	U
87	U	U	U	U	U	U	U	U	U	U
88	U	U	U	U	U	U	U	U	U	U
89	U	U	U	U	U	U	U	U	U	U
90	U	U	U	U	U	U	U	U	U	U
91	U	U	U	U	U	U	U	U	U	U
92	U	U	U	U	U	U	U	U	U	U
93	U	U	U	U	U	U	U	U	U	U
94	U	U	U	U	U	U	U	U	U	U

95	U	U	U	U	U	U	U	U	U	U
96	U	U	U	U	U	U	U	U	U	U
97	U	U	U	U	U	U	U	U	U	U
98	U	U	U	U	U	U	U	U	U	U
99	U	U	U	U	U	U	U	U	U	U
100	U	U	U	U	U	U	U	U	U	U
101	U	U	U	U	U	U	U	U	U	U
102	U	U	U	U	U	U	U	U	U	U
103	U	U	U	U	U	U	U	U	U	U
104	U	U	U	U	U	U	U	U	U	U
105	U	U	U	U	U	U	U	U	U	U
106	U	U	U	U	U	U	U	U	U	U
107	U	U	U	U	U	U	U	U	U	U
108	U	U	U	U	U	U	U	U	U	U
109	U	U	U	U	U	U	U	U	U	U
